# Psychometric evaluation of Persian version of Diabetes Acceptance Scale (DAS)

**DOI:** 10.1186/s12902-022-01123-2

**Published:** 2022-09-08

**Authors:** Tahereh Najafi Ghezeljeh, Hamid Sharif Nia, Hossein Bagheri, Ali Abbasi, Sahar Keyvanloo Shahrestanaki, Hossein Amiri Largani, João Marôco

**Affiliations:** 1 Nursing Care Research Center, School of Nursing and Midwifery, Iran University of Medical Sciences, Tehran, Iran; 2 Amol Faculty of Nursing and Midwifery, Traditional and Complementary Medicine Research Center, Addiction Institute, Mazandaran University of Medical Sciences, Sari, Iran; 3 Department of Nursing, School of Nursing and Midwifery, Shahroud University of Medical Sciences, Shahroud, Iran; 4 Student Research Committee, School of Nursing and Midwifery, Iran University of Medical Sciences, Tehran, Iran; 5 School of Medicine, Shahroud University of Medical Sciences, Shahroud, Iran; 6 William James Centre for Research, ISPA – Instituto Universitário, Lisbon, Portugal

**Keywords:** Diabetes mellitus, Acceptance, Psychometric, Validity, Reliability

## Abstract

**Background:**

Acceptance of diabetes is a psychological adaptation to the potential limitations of the disease. Poor acceptance of diabetes impairs effective self-management of diabetes, leading to worsening metabolic control. This study aimed at determining the psychometric properties of the Persian version of the Diabetes Acceptance Scale.

**Methods:**

This cross-sectional methodological study was performed on diabetic patients in Iran in 2021. The questionnaire consisted of two parts: demographic characteristics and Diabetes Acceptance Scale. The questionnaire was translated into Persian through the forward–backward translation method. The face validity and content validity were performed qualitatively and quantitatively. Exploratory (*n* = 200) and confirmatory (*n* = 200) factor analysis were performed to evaluate the validity of the structure. Internal consistency and temporal stability were estimated to determine reliability.

**Results:**

Exploratory factor analysis on the polychoric correlation matrix obtained three factors: Rational dealing, Resentment and Avoidance, which explained 68.8% of the total DAS variance. Confirmatory factor analysis showed that the 3-fractor model had a good fit to a second independent data set. Finally, Ordinal Cronbach's alpha coefficient was 0.96, 0.94 and 0.93, respectively for the Rational dealing, Resentment, and Avoidance factor. Also, using intraclass correlation coefficient, the stability of the instrument was 0.97.

**Conclusion:**

Based on the findings of this study, the Persian version of DAS has sufficient validity and reliability to measure the admission of Iranian diabetic patients.

## Background

Diabetes is a common metabolic disease in the world. By 2019, about 463 million adults were suffering from diabetes, 79% of whom were from low- and middle-income countries. If the current trend continues, these figures are expected to increase to 700 million by 2045 [1]. In Iran, 11.9% of 25 to 70 years old adults have been reported to suffer from diabetes (2011), which shows an increase of 35% compared to 2005. It is estimated that by 2030, about 9.2 million Iranians may suffer from diabetes [2]. The increase in the incidence of this disease is associated with an increase in morbidity and mortality. Therefore, the goal of diabetes treatment is to reduce mortality and prevent complications [3]. In fact, following treatment can improve blood sugar control and help patients prevent long-term complications of diabetes [4].

Acceptance of a chronic disease like diabetes is of great importance in the proper management of this disease. This acceptance will lead to changes in lifestyle and ways of self-care and will make it easier for diabetics to continue treatment and care [5]. The extent to which a person accepts the disease as part of their life and integrates its psychosocial impact and emotional burden is known as "diabetes acceptance" [6]. Diabetes acceptance is a psychological adaptation to the potential limitations that typically accompany a chronic condition and can lead to control, cohesion, openness, care [7], and ultimately, integrating a chronic condition into one's lifestyle [8]. However, non-acceptance of the disease leads to surrender to the imposed limitations by that disease, reduced self-sufficiency and self-confidence, and feelings of dependence on others [9]. Generally, accepting of chronic disease is a fundamental issue and does not correspond to feeling weak against the disease and denying it, but it is rooted in self-confidence to accept and cope with issues out of control and helps the patient to tolerate the disease better. Non-acceptance may cause non-compliance with the disease and delay the treatment process [10].

Proper adaptation to diabetes usually leads to maintaining or improving one's integrity (i.e., a sense of self-sufficiency). However, low acceptance of diabetes can be described by dysfunctional attitudes and behaviors such as incompatibility, avoidance, and suppression of the disease, which may indicate defense strategies to prevent devaluation [11, 12]. Low diabetes acceptance is also associated with decreased self-management and higher HbA1c in both diabetic groups of adults [13–15] and adolescents [16, 17]. Furthermore, poor adaptation to diabetes has been suggested as a risk factor for emotional distress [18, 19]. Thus, it may hurt life quality of patients and, potentially, increase the risk of depression associated with diabetes [20]. Accordingly, several studies have reported evidence of the low quality of life [9, 21], and a high prevalence of depression and distress [13, 22, 23] in patients with low diabetes acceptance. Schmitt et al. (2014) suggest that assessing diabetes acceptance can help identify patients with inadequate self-care and poor diabetes control [13].

Despite the high clinical significance of this issue, there are limited research and understanding in this area. According to Schmitt et al. (2018), lack of research is probably due to the limited number and lack of appropriate measurement and satisfactory instruments, respectively [24]. Some studies have used general classical criteria for disease acceptance, such as the Acceptance of Illness Scale (AIS) or the Acceptance of Disability Scale (ADS) [9, 14, 15]. Although these tools may seem valid, they are not specific to diabetic patients, it is preferable to use a specific scale to measure diabetes acceptance.

Gregg et al. Designed the Acceptance and Action Diabetes Questionnaire (AADQ) as a dedicated instrument for non-acceptance of diabetes [22]. In this regard, Schmitt et al. (2018) stated "we found evidence of psychometric limitations of the scale (i.e. suboptimal properties and fitting of five of the eleven items). Moreover, since the AADQ was developed by simply adapting items from a generic experiential avoidance measure towards diabetes, 25 a more systematic development approach (i.e., theory-driven item design and empirical selection) might yield a more effective instrument” [24]. Also, the 42-item Diabetes Acceptance and Action Scale (DAAS), developed by Berlin et al. focuses on the rejection of diabetes only in patients with type 1 diabetes [25].

Although several general and specific measuring instruments have been introduced that can assess the acceptance of diabetes, DAS is a suitable and specific instrument for measuring adult diabetes acceptance. Schmitt et al. (2018) designed a dedicated instrument called the Diabetes Acceptance Scale (DAS) and described its clinical application. They also stated that DAS is a valid instrument for measuring diabetes acceptance. This instrument may help research the psychological factors that influence diabetes control and predict health outcomes. It is also useful in clinical care to evaluate the results of behavioral therapies for patients with diabetes control problems [24]. DAS is a new and dedicated instrument, the design and psychometrics of which have been done by Schmitt et al. (2018). In Iran, due to the lack of specific tools to measure the diabetes acceptance in adults with type 1 and type 2 diabetes mellitus, psychometrics of this instrument and its application in the future can be justified. Nurses and health professionals in Iran cannot have easy and low-cost access to specialized instruments for evaluating diabetes acceptance. Therefore, this study aimed at translating and determining the psychometrics of diabetes acceptance instruments, so that it might play a significant role in better diabetes control and effective clinical care.

## Materials and methods

This cross-sectional methodological study was performed among diabetic patients referred to the diabetes clinic and hospitals affiliated with Shahroud University of Medical Sciences from March to July 2021.

### The scale

The 20-item Diabetes Acceptance Scale is based on a four-point Likert scale from "never true for me" (0) to "always true for me" (3). A higher scale indicates higher diabetes acceptance. This scale includes 10 positive items (including acceptance, integration, and identification—items 1 to 10) on the one hand and 10 negative items (including non-acceptance, avoidance, and neglect—items 11 to 20) on the other. The total scale of this instrument varies from 0 to 60 and scales greater than or equal to 30 indicate high acceptance of the disease [24].

### Translation

Initially, the questionnaire was purchased and permission to use it was obtained from Professor Schmitt (Appendix 1). Then it was translated according to the translation protocol of WHO [26] through the forward–backward method. Thus, two independent experts translated the questionnaire from English to Persian. After that, an integrated Persian version of the above two translations was presented. Subsequently, two experts translated the Persian version into English and compared it with the original English version by the research team, which was sent to Professor Schmitt for final approval and was approved by him. Finally, all comments are included in the final version of this scale. There was also no significant cross-cultural change in scale.

### Face validity

For the qualitative face validity stage, the scale was given to ten patients with type 1 and 2 diabetes. Their views on appropriateness, difficulty, relevancy, and ambiguity were assessed through interviews.

For the quantitative face validity stage, ten diabetic patients were asked to rate the importance of each item on a 5-point Likert scale (5 = very important, 4 = important, 3 = almost important, 2 = slightly important, 1 = not important). The impact score of the items was obtained from the following formula: Impact score = Frequency (%) * Importance.

The item impact score was calculated by multiplying the frequency in the form of a percentage (the number of people who scored 4 and 5 to each item) by the importance (mean score of importance based on the Likert scale). An impact score > 1.5 was considered appropriate for each item [27, 28].

### Content validity

In order to evaluate the content validity, 12 experts (two clinical psychologists, a psychiatrist, two diabetes endocrinologists, five nursing assistant professors, and two experienced assistant professors in questionnaire design) were interviewed to qualitatively review the questionnaire and give comments on observing grammar, using appropriate words, placing items in their proper place, and the scaling of the questionnaire. The experts did not make any changes to the questionnaire.

Quantitative content validity was assessed by calculating the content validity ratio (CVR) and content validity index (CVI) for items. The same 12 experts were asked to validate the content and assess the essentiality of the items as follows: 1: not necessary, 2: useful but not necessary, 3: necessary [29].

CVR was calculated by the following formula:

CVR = (*ne*—[ *N* / 2]) / ( *N* / 2).

In this formula, *N* and *ne* are equal to the total number of experts and the number of experts who rank the item as "necessary", respectively. Considering the number of experts that is 12, the minimum acceptable CVR is 0.56 [30].

The CVI shows the relevance of the items on the scale. It can be calculated for each scale item (I-CVI) as well as for all items (S-CVI). Therefore, we asked the same 12 experts to review the items based on the "not related", "somewhat relevant", "relevant but in need of review" and "fully relevant" options and give 1, 2, 3, and 4, respectively.

The CVI of each item was calculated by dividing the number of participants who gave 3 or 4 to that item by the total number of participants. Item acceptance was based on a CVI above 0.79 being considered adequate. The score between 0.70 to 0.79 was considered questionable and needed to be corrected and revised. Also, a less than 0.70 score was considered unacceptable and should be removed [31].

### Participants and the study setting

Based on the Rule of Thumb that considers 200 participants as the adequate sample size [32], at least 200 diabetic patients (10 participants per item) were required for exploratory factor analysis (EFA) (sample 1) and similarly 200 diabetic patients (sample 2) for confirmatory factor analysis (CFA) [33]. A total of 400 patients with type 1 and 2 diabetes from internal and endocrinology wards and specialized diabetes clinics affiliated with Shahroud University of Medical Sciences were selected based on inclusion criteria and convenience sampling method. Data were collected via pen and paper method by referring to these centers.

The Inclusion criteria in this study were: having type 1 and 2 diabetes diagnosed by a physician, being over 18 years old, being literate, and having fluency in Persian.

### Construct validity

The construct validity of DAS was evaluated by Exploratory Factor Analysis on the Polychoric correlation matrix using the psych package [34, 35] with the Kaiser criterium. An initial test sample of *n*=200 participants was used.

Kaiser–Meyer–Olkin (KMO) test and Bartlett test were performed for adequacy sampling. KMO values between 0.7 and 0.8 were considered good and values between 0.8 and 0.9 were considered excellent [36, 37]. The presence of an item in a latent factor was determined based on a factor loading of approximately 0.33, which was estimated using the following formula: CV = 5.152 ÷ √ (*n* – 2) in which CV was the number of extractable factors and n was the sample size. Next, item subsets less than 0.3 in size were removed from the EFA [38].

CFA was used to evaluate the most common goodness of fit indices for the proposed model concerning acceptable thresholds using maximum likelihood estimation. Confirmatory Factor Analysis (CFA) was performed on the polychoric correlation matrix of the ordinal items on a second independent sample from the one used in EFA using the DWLS (Diagonally Weighted Least Squares) implemented in the lavaan package [39] for the R Statistical System.

The model fit was examined with Root Mean Square of Error of Approximation (RMSEA < 0.08), Square root mean residual (SRMR < 0.08), Comparative Fit Index (CFI > 0.9), Tucker-Lewis Fit Index (TLI > 0.9). [40, 41]. Three different models were fit: the first one with the three first order factor obtained from the EFA (model 1), the second model with a second order factor (acceptance of diabetes) reflected in the three first order factors, and a third bi-factor model where a general acceptance of diabetes factor was added orthogonally to three first order factors extracted with the EFA. The goodness of fit for the three models were compared with a Scaled Chi square difference test using the Satorra method. The lavaan package for the R statistical system was used for CFA and model comparison [39].

### Convergent and discriminant validity

Convergent and discriminant validity of DAS was calculated using Fornell and Larcker's approach and through Average Variance Extracted (AVE), Maximum Shared Squared Variance (MSV), and Composite reliability (CR). AVE greater than 0.5 indicated appropriate convergent validity and AVE greater than MSV confirm discriminant validity [42]. Hair et al. states that convergent validity exists when the items of the construct are close to each other and have high shared variance. Also, divergent validity exists when the items of the construct or the latent extracted factors are completely apart from each other [43]. In the clearer sense, if the latent factors cannot be explained by the extracted causes and are not sufficiently correlated, we won’t have proper convergent validity [42].

### Reliability

In order to evaluate the internal consistency of DAS, Cronbach's alpha coefficient and McDonald’s omega (Ω), was estimated for each extracted factor. An alpha value and Ω values above 0.7 indicated good internal consistency [40]. The construct reliability (CR) was then examined for each factor. CR values greater than 0.7 indicated good reliability [33].

The stability was assessed by the intraclass correlation coefficient (ICC). The index higher than 0.75 indicated good stability of the scale [44]. A sub-sample of 20 diabetic patients completed the questionnaire twice with an interval of two weeks. SPSS and Amos version 26.0 were used for statistical analysis.

### Normality, outliers and missing data

Univariate and multivariate outliers were evaluated using distribution charts and Mahalanobis distance *p* < 0.001 respectively. Furthermore, univariate normality and multivariate normality distributions were checked by skewness (values within ± 3) and kurtosis (values within ± 7) and Mardia's coefficient < 8 respectively. In the present study, the data did not deviate strongly from the normal distribution. We used a listwise missing procedure for CFA estimation. We prefer listwise deletion instead of imputation because missingness was associated with non-responses and incomplete questionnaires.

## Results

A total of 400 diabetic patients participated in this study. Out of this number, 225 (56.2%) were female, 81% (*n* = 324) were married and 176 (44%) were housewife. Also, 12.2% and 87.8% suffered from type 1 and type 2 diabetes, respectively. The characteristics of the participants are shown in Table 1.

**Table 1:** The characteristics of study participants (*n* = 400)

Variables		Number (%)
**Gender**	Male	175 (43.8)
	Female	225(56.2)
**Educational**	Primary	141 (35.5)
	Secondary	185 (46)
	Higher	74 (18.5)
**Marital status Employment status**	Married	324 (81)
	Single	20 (5)
	Widowed	42 (10.5)
	Divorced	14 (3.5)
	Housewife	176 (44)
	Employed	93 (23.3)
	Retired	117 (29.2)
	Unemployed	14 (3.5)
**Adequacy of income**	Yes	161 (40.2)
	No	239 (59.8)
**Type of diabetes**	type 1	49 (12.2)
	type 2	351 (87.8)
		**Mean (SD)**
**Age (years)**		56.43 (12.6)
**Diabetes duration (years)**		9.37 (6.87)
**HbA1c (%)**		8.05 (1.84)

### Face and content validity

According to the results of face validity, all items of the questionnaire were appropriate, clear, and important. Also, the results of quantitative face validity showed that all scores were above 1.5 and none of the items were removed.

After conducting content validity and applying experts' opinions, out of 20 initial items related to the scale, item number 2 was removed due to a negative CVR (-0.16), and the number of scale items reached 19.

### Construct validity

In EFA, the KMO value was 0.879, and Bartlett’s test of sphericity was 9837.191 (P < 0.001). In the model, three factors were extracted. Horn’s parallel analysis and the Kaiser criterium were used for extracting factor structure using the psych package on the polychoric correlation matrix. As shown in Table 2, the three factors together accounted for 68.8% of the total variance.

**Table 2:** Exploratory Factors analysis of the Diabetes Acceptance Scale (*n*=200)

Factors	Qn. Item	Factor Loading	Eigenvalue	%Variance
**Rational Dealing**	**8. I integrate diabetes in my daily routine as much as possible.**	**0.945**	**11.03**	**40.0**
	**3. Diabetes is a normal part of my life.**	**0.805**		
	**9. I fully accept living with diabetes.**	**0.934**		
	**4. Living with diabetes is a part of me.**	**0.733**		
	**6. I accept diabetes as a part of my life.**	**0.893**		
	**1. I have accepted diabetes.**	**0.786**		
	**7. I’m motivated to treat my diabetes properly.**	**0.950**		
	**10. I take a good care of my diabetes.**	**0.896**		
	**5. I ensure that my diabetes treatment works well.**	**0.834**		
	**14. I refuse accepting diabetes as a part of my life.**	**0.468**		
	**19. I have difficulty in motivating myself to perform appropriate diabetes self- care.**	**0.587**		
**Resentment**	**12. When I think of living with diabetes, I feel low / depressed.**	**0.857**	**2.86**	**16.8**
	**11. I suffer from having diabetes.**	**0.853**		
	**18. Living with diabetes makes me sad / depressed.**	**0.888**		
	**15. Diabetes causes dissatisfaction in my life.**	**0.731**		
**Avoidance**	**16. I avoid dealing with topics related to diabetes.**	**0.759**	**1.05**	**11.7**
	**17. I avoid things that remind me of diabetes.**	**0.710**		
	**20. I neglect diabetes self-care because I want to avoid diabetes-related topics.**	**0.533**		
	**13. I often ignore diabetes.**	**0.586**		

### Confirmatory factor analysis

**Fig. 1:**
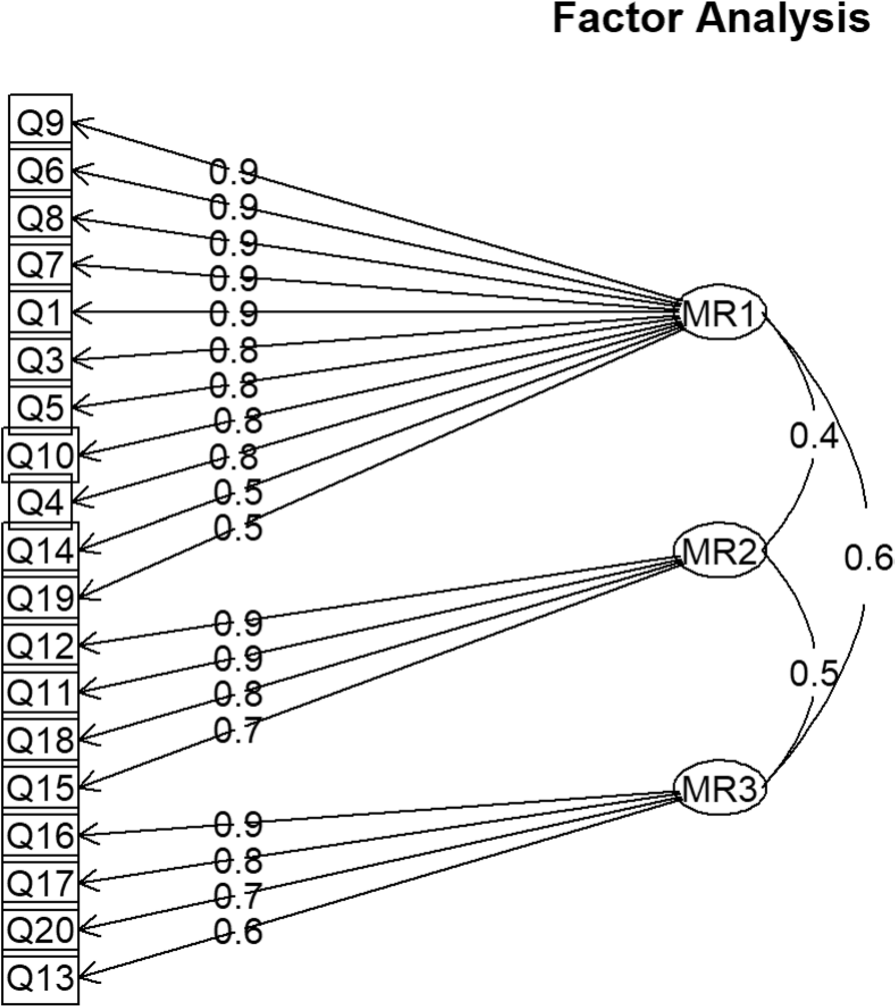
The final model of Diabetes Acceptance Scale (MR1 -Rationale Dealing; MR2 –Resentment; MR3 – Avoidance)

Fit for the three models described previously is presented Table 3. Both model 1 (three first order factors; Fig. 1) and model 2 (second order factor reflected on the three first order factors) displayed a good and equivalent fit to the data (see Table 3).

**Table 3:** Goodness of fit for the three CFA models tested

Model	Goodness of fit Indices				
	χ^2^( df)	CFI	TLI	SRMR	RMSEA
1 – Three first order factors	959.9 (148)	0.956	0.949	0.084	0.117
2 – Second order factor	959.9 (148)	0.956	0.949	0.084	0.117
3 -- Bi-factor model	4062.8 (133)	0.786	0.725	0.165	0.272

The Scaled Chi-Squared Difference Test showed that there were no differences in the goodness of fit for models 1 and 2 (χ^2^(148) = 887.65, *p* < 0.001). but a significant worse fit was displayed by model 3 (bi-factor) as compared to models 1 or 2(Δχ^2^(15) = 1095.9, *p* = 0.999).

### Convergent and discriminant validity

Since AVE was greater than 0.5, it indicated appropriate convergent validity, and the discriminant validity was confirmed due to AVE greater than MSV (Table 4).

**Table 4:** Convergent and Discriminant Validity, and Reliability of the DAS (*n*=200)

Indices	CR	AVE	MSV	MaxR (H)	α_**ordinal**_	Ω	ICC
Factor							
**Rational Dealing**	0.944	0.608	0.304	0.952	0.960	0.948	0.971
**Resentment**	0.901	0.695	0.286	0.908	0.944	0.913	0.965
**Avoidance**	0.873	0.636	0.304	0.907	0.925	0.808	0.926

### Reliability

Cronbach's alpha (α), McDonald’s omega (Ω), and ICC were two excellent factors derived from DAS (Table 4). The ICC of all items was calculated to be 0.974. Also, CR above 0.7 showed good construct reliability.

## Discussion

Based on EFA, the results of this study showed three factors in DAS: Rational dealing, Resentment, and Avoidance, that total of 19 items of this scale explained 68.8% of the total variance. The original version of DAS (20 items) had a single-factor structure and was designed to overcome the limitations of the previous diabetes acceptance instrument [24]. In a similar study, Schmitt et al. (2016) designed a 28-item version of DAS that differs from the original version of the present study scale. The 28-item version of DAS included four factors: integration, motivation, avoidance/defense, and distress [45]. However, the original version of DAS was a single-factor and 3 factors were extracted in the EFA of the Persian version and confirmed with the CFA.

Illness acceptance can be broadly defined to as the successful adjustment to a chronic illness and to the unpredictable and adverse consequences associated with the disease [46]. In this regard, Zheng et al. (2019) stated that understanding of illness; overcoming limitations, normalization, and readiness for responsibility are four key attributes of illness acceptance [47]. In this study, according to the culture of diabetic patients in Iran, this concept was extracted with three factors of rational dealing, resentment, and avoidance. The first extracted factor from the Persian version of DAS was the rational dealing in eleven items, which emphasized acceptance and adaptation to diabetes. This factor explained the highest percentage of diabetes acceptance and showed its importance in accepting diabetes. In this factor, 9 items were positive and two items was negative. In psychometrics analysis of the 28-item version of DAS, Schmitt et al. (2016) explained the two factors of integration and motivation [45] and it was equivalent to rational dealing in the present study. Psychological adaption to diabetes can be considered as a growing trend toward acceptance, which includes tolerance, adaption, integration, and identification [18].

The second extracted factor from the Persian version of DAS was resentment in four items, all of which were negative and focused more on the pain caused by diabetes, frustration, sadness and dissatisfaction with diabetes. Finally, the third extracted factor from the Persian version of DAS was Avoidance in four items, all of which were negative and included ignoring diabetes, refraining from talking about diabetes, avoiding reminding of diabetes, and neglecting self-care due to staying away from diabetes-related topics. As mentioned above, EFA extracted 4 factors in psychometrics of the 28-item version of DAS, of which the distress factor indicated diabetes-induced problems in patients’ lives and the avoidance/defense factor indicated avoiding any diabetes-related issues [45]. Resentment and avoiding were the two factors extracted in this study that indicated the non-acceptance of diabetes. In this regard, low acceptance of diabetes can be described as hostility, avoidance, denial, or neglect of the disease. Therefore, it is reasonable to assume that poor acceptance of diabetes may impair effective self-management of diabetes and thereby lead to poor metabolic control of the disease [13, 48].

Since all fit indices of this study (model 1 and 2) were in the acceptable range at the time of performing CFA, the model had a good fit with the data and all the indicators were satisfactory. In the psychometrics analysis of the original version of DAS, Schmitt et al. (2018) evaluated factor validity by CFA, which indicated good factor validity. According to CFA of a single-factor structure which indicated a good fit, (SRMR = 0.02; TLI = 0.99; CFI = 0.99; RMSEA = 0.03, 90% CI]0.02; 0.04[ [24]. Also, in psychometrics of the 28-item version of DAS, Schmidt et al. (2016) supported CFA of the four-parted scale structure (SRMR = 0.049, CFI = 0.936, RMSEA = 0.066) [45]. As previously stated, in this study there were no differences in the goodness of fit for models 1 and 2, but a significant worse fit was displayed by model 3 (bi-factor) as compared to models 1 or 2. These results showed that the diabetes acceptance is identified in three factors, not one factor.

The results of this study showed that DAS items of the final model have good convergent and discriminant validity. In this regard, there was a good convergent validity between DAS and AADQ in the study of Schmitt et al. (2018). Also, the observed association between DAS and variables that were expected to be associated with diabetes acceptance (such as coping, emotional distress, self-management, and diabetes outcomes) showed the criterion validity of the original version of the scale [24]. Also, the 28-item version of DAS showed good convergence with the parallel criterion of non-acceptance of AADQ (r = -0.65, *P* < 0.001) [45].

In this study, Cronbach's alpha coefficient and McDonald’s omega for each of the factors was acceptable because alpha and omega were higher than 0.7 [33]. Therefore, the results indicated desirable the internal consistency of the items in the Persian version of DAS. Also, the CR scale was evaluated by CFA and since it was above 0.7 in all factors, it indicated the appropriate construct reliability. One of the advantages of CR measurement is that this estimate is not affected by the number of scale items and the obtained structure and it depends on the actual factor load of each item of latent variables [49]. In line with this finding, Schmitt et al. (2018) also showed that Cronbach's alpha coefficient for the whole scale in the original version was 0.96 [24]. In a similar study, the 28-item version of DAS showed high reliability (Cronbach's alpha: 0.96 overall scale; subscales: integration 0.94, motivation 0.92, avoidance/defense 0.89, distress 0.89) [45].

The stability was assessed by retest analysis. Significant correlations were obtained from the first and second evaluations. This finding supported the temporal stability of the Persian version of DAS. Bond et al. (2011) obtained the reliability of AAQ-II by 0.81 and 0.79 through 3-month and 12-month retests, respectively, which indicated the good stability of AAQ-II [50].

Since numbers 2 item were removed in the Persian version of this scale, thus the total score of the instrument varied from 0 to 57 and higher scores indicated more acceptance. The Persian version of DAS includes three factors: Rational dealing in eleven items (scores 0 to 33), Resentment in four items (scores 0 to 12) , and Avoidance in four items (scores 0 to 12).

### Limitation

Data collection was only in two cities of Iran. Thus, the generalizability of the study may be a limitation. In addition, besides Iran, other countries also speak Persian. Using this questionnaire in these countries should be adapted to their culture.

## Conclusions

According to the findings of this study, DAS in Iranian diabetic patients consists of 19 items and three factors that explain more than half of the variance of this concept. DAS has good reliability, internal consistency, and construct validity for identifying "diabetes acceptance". DAS is a simple, practical, and valid instrument to evaluate the acceptance of diabetic patients and can play an important role in better control of diabetes and effective clinical care. Since this instrument has been validated in Iran, it can lead to the development of its use in research and care and treatment clinics to reduce the problems of diabetic patients.

## Data Availability

The datasets used and/or analyzed during the current study are available from the corresponding author on reasonable request.

## Abbreviations

DAS: Diabetes Acceptance Scale
AIS: Acceptance of Illness Scale
AADQ: Acceptance and Action Diabetes Questionnaire
DAAS: Diabetes Acceptance and Action Scale
CVR: Content validity ratio
CVI: Content validity index
I-CVI: Item content validity index
S-CVI: Scale content validity index
EFA: Exploratory factor analysis
CFA: Confirmatory factor analysis
DWLS: Diagonally Weighted Least Squares
KMO: Kaiser–Meyer–Olkin
RMSEA: Root Mean Square of Error of Approximation
SRMR: Square root mean residual
CFI: Comparative Fit Index
TLI: Tucker-Lewis Fit Index
AVE: Average Variance Extracted
MSV: Maximum Shared Squared Variance
CR: Composite reliability
ICC: Intraclass correlation coefficient
